# Controllable Fabrication of Percolative Metal Nanoparticle Arrays Applied for Quantum Conductance-Based Strain Sensors

**DOI:** 10.3390/ma13214838

**Published:** 2020-10-29

**Authors:** Zhengyang Du, Ji’an Chen, Chang Liu, Chen Jin, Min Han

**Affiliations:** National Laboratory of Solid State Microstructures, College of Engineering and Applied Sciences and Collaborative Innovation Centre of Advanced Microstructures, Nanjing University, Nanjing 210093, China; mg1834021@smail.nju.edu.cn (Z.D.); dg1934012@smail.nju.edu.cn (J.C.); dg1634023@smail.nju.edu.cn (C.L.); jinchen_1992@smail.nju.edu.cn (C.J.)

**Keywords:** nanoparticle arrays, gas phase deposition, percolation, quantum conductance, strain sensors

## Abstract

We use gas phase deposition of well-defined nanoparticles (NPs) to fabricate closely-spaced Pd NP arrays on flexible membranes prepatterned with interdigital electrodes (IDEs). The evolution of the morphology and electron conductance of the NP arrays during deposition is analyzed. The growth of two-dimensional percolation clusters of interconnected NPs, which correlate with the percolation pathway for electron conduction in the NP deposits, is demonstrated. The percolative nature of the NP arrays permits us to finely control the percolation geometries and conductance of the NP film by controlling the NP deposition time so as to realize a precise and reproducible fabrication of sensing materials. Electron transport measurements reveal that the electrical conductance of the NP films is dominated by electron tunneling or hopping across the NP percolating networks. Based on the percolative and quantum tunneling nature, the closely-spaced Pd NP films on PET membranes are used as flexible strain sensors. The sensor demonstrates an excellent response ability to distinguish tiny deformations down to 5×10^−4^ strain and a high sensitivity with a large gauge factor of 200 up to 4% applied strain.

## 1. Introduction

Strain and pressure sensors are important micro-electro-mechanical system (MEMS) devices, with applications in various areas such as industrial control, automotive and aerospace systems, consumer electronics, environmental monitoring and wearable devices and electronic skins [1,2,3,4]. To achieve strain and pressure gauging, a number of transduction mechanisms including capacitive, piezoelectric, piezoresistive, as well as transistor sensing have been utilized. Among them piezoresistive sensing is the most frequently used transduction mechanism, owing to advantages such as low cost, scalable, DC input and easy signal collection, as well as its simple structure and manufacturing process [5,6,7].

However, conventional resistive strain sensors, mainly metallic foil or semiconductor strain gauges, show significant limitations, such as the low gauge factor of the former or the fragile and high temperature coefficient of the latter. Recently, conductive polymer composites incorporating conductive fillers such as carbon blacks, nanoparticles (NPs), nanotubes, as well as graphene membranes in/on an insulating polymer matrix have been used as strain/pressure sensing elements [8,9,10,11,12,13,14,15,16]. Strains applied on these composites may cause a drastic fall in resistivity due to the formation of percolation paths [15]. However, the sensitivities of the devices based on such materials are moderate and susceptible to significant hysteresis. Furthermore, the uncontrollable, sudden drastic resistivity drop at the critical volume fraction and the non-reliability (large variations) on the electric response induce many problems regarding fabrication and assessment.

Recently, a new configuration of piezoresistive strain/pressure sensing elements fabricated from percolation-based conductive closely-spaced metal NP arrays was proposed [17,18,19]. Electron conduction in the highly disordered NP arrays is dominated by a Coulomb blockade and electron tunneling and hopping across the NP percolating networks [20,21,22]. The conductance of the NP arrays deposited on the electrodes is correlated to the number of percolative paths they contain. Since electron conduction by tunneling or hopping between closely-spaced NPs is extremely sensitive to inter-particle spacing, breaking or regenerating of the percolative paths could be induced by tiny changes in the NP array geometries. Differently from current piezoresistive strain sensors, the devices based on the closely-spaced NP arrays transduce the deformation on the electrodes covered with NPs to the change of the quantum conductance of the NP arrays [17,23]. They are characterized with high sensitivities and resolutions, wide dynamic ranges, reduced thermal disturbances, as well as reduced power consumptions.

In this paper, we fabricate closely-spaced metallic NP films via gas phase depositing of preformed NPs with a controlled coverage. The evolution of the electron conductance and the geometry of the NP percolating networks with the deposition process and the deformation of the flexible substrate is analyzed. The closely-spaced metal NP films are employed to fabricate a strain sensor prototype, and its strain sensing characteristic is demonstrated.

## 2. Materials and Methods

Closely-spaced metal NP films were prepared with gas phase NP deposition. Generally, NPs of either metals could be used in the present piezoresistive sensing scheme. Here, we chose Pd NPs since they hardly coalesce after depositing on the surface even at a very high NP density so that prefect percolative NP arrays can be formed. This behavior is important to achieve a high stability in sensor applications.

**Figure 1:**
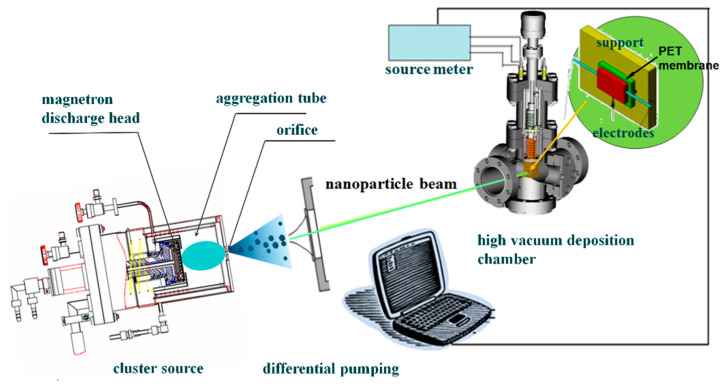
A schematic diagram depicting the cluster source and nanoparticles (NP) beam deposition setup.

A Pd NP beam was generated in a magnetron plasma gas aggregation cluster source [24,25]. The setup consisted of a DC magnetron discharge head equipped in an aggregation tube ended with an orifice 3 mm in diameter, a differential pumping unit, as well as a high vacuum deposition chamber. Figure 1 shows a schematic drawing of this setup. A planar Pd target of 99.999% purity was used for magnetron sputtering. 140 sccm of argon flow (99.99% purity) was introduced into the aggregation tube to maintain a stable pressure of 120 Pa. The magnetron discharge was operated by a 500 W power supply (MDX500, Advanced Energy, Fort Collins, CO, USA). Pd atoms were sputtered from the target and formed clusters through the aggregation process in the argon gas. By extracting the clusters at the high vacuum differential pumping stage through the orifice, a collimated NP beam was formed. The Pd NPs were deposited on the substrate in the high vacuum deposition chamber. A stable deposition rate of 0.4 Å/s, monitored using a quartz crystal microbalance, was maintained by regulating the discharge power. By controlling the deposition time with a shutter, the coverage of NPs on the substrate could be precisely controlled.

To fabricate strain sensors and measure the conductance of the closely spaced NP arrays, Pd NPs were deposited on interdigital electrodes (IDEs) prepatterned on the surface of polyethylene terephthalate (PET) membrane. The PET membrane is a 6 × 20 mm^2^ slice with a thickness of 0.1 mm. The silver IDEs, with a 100 nm thickness and 15 μm electrode separation, were deposited on the membrane via shadow mask vacuum evaporation. The electrodes covered an area of several ten square millimeters, with an as-prepared resistance of about 10^10^ Ω. During NP deposition, the conductance across the electrode gap was monitored in real time. The deposition was cut immediately by operating a shutter when a predetermined conductance was approached.

The morphologies of the Pd NP arrays were characterized with a transmission electron microscope (TEM, FEI TECNAI F20s TWIN, Thermo Fisher Scientific, Hillsboro, OR, USA). For TEM characterization, amorphous carbon films supported on copper grids were used as substrates. The TEM images were digitalized by using a public domain Java image processing program, ImageJ (National Institutes of Health). The sizes and locations of each nanoparticle were extracted. A Visual Basic program written by ourselves was used to perform percolation analysis on the digitalized TEM images.

The conductance and current-voltage (I-V) characteristic of the NP arrays were measured with a digital source meter (Keithley 2400). The data acquisition was carried out with a LabView coded program through a USB interface. The temperature of the sample was constantly controlled in the range of 10–300 K by thermally contacting the sample with the copper finger of an optical cryostat system (Janis CCS-150, Janis Research, Wilmington, MA, USA).

The response characteristics of the fabricated strain sensors were evaluated by measuring the conductance changes of the NP arrays deposited on the IDEs following the applied strains. The strains were induced by subjecting the PET membranes to a series of bending cycles or pull-and-release cycles driven with a micrometer. In the bending case, the PET membrane was deformed by adjusting the micrometer getting in touch with the membrane surface near the location of the IDEs. The strain was calculated from the deformation geometry of the membrane surface measured from a microscope. In the pull-and-release case, one side of the PET slice was fastened on the fixed base of an optical bench adjusting bracket, and the other side was pulled with the micrometer of the adjusting bracket. The strain was calculated directly from the relative change of the length of the PET slice reading from the micrometer. The conductance was measured by recording the current in the NP arrays, with a constant bias voltage of 1 V applied to the IDEs.

## 3. Results and Discussion

**Figure 2:**
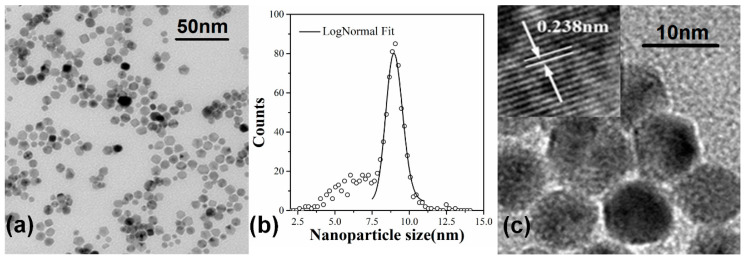
(**a**) A transmission electron microscope (TEM) microimage of the deposited Pd NP arrays showing a closely spaced assembling morphology. (**b**) Size distribution histogram of Pd NPs measured from the TEM image. (**c**) A high-resolution TEM image demonstrating polyhedron-shaped Pd NPs arranged in close proximity.

Figure 2a shows a TEM microimage of a film of the deposited Pd NPs. The NPs are randomly distributed and well isolated. The film contains numerous closely spaced NP assembling areas. The size distribution histogram of the Pd NPs is counted from the TEM image and shown in Figure 2b. We can see the NP assemblies are mainly composed of a group of NPs with diameters between 7.5 nm to 10 nm. Their size distribution can be well fitted with a log-normal function with a mean diameter of 9 nm and a standard deviation of about 1.4 nm. Meanwhile, a small number of smaller NPs can also be observed, with a broad size distribution from 2.5 nm to 7.5 nm. In addition, a tiny number of larger NPs with diameters around 12.5 nm can be distinguished. They may coalesce from smaller ones after depositing on surface. From the high resolution TEM image shown in Figure 2c, flat facets can be frequently identified from the individual Pd NPs, indicating that the NPs are polyhedron nanocrystals with random orientations. In the assembling region, although the adjacent NPs are in close proximity to each other, it can be distinguished in the TEM image that the NPs are separate from each other with an edge-to-edge distance of about 1 nm, sometimes 1.5 nm or more. The gaps separating the NPs might be thin amorphous shells, most likely PdO_x_, developed on the NP surfaces due to oxidation [15].

**Figure 3:**
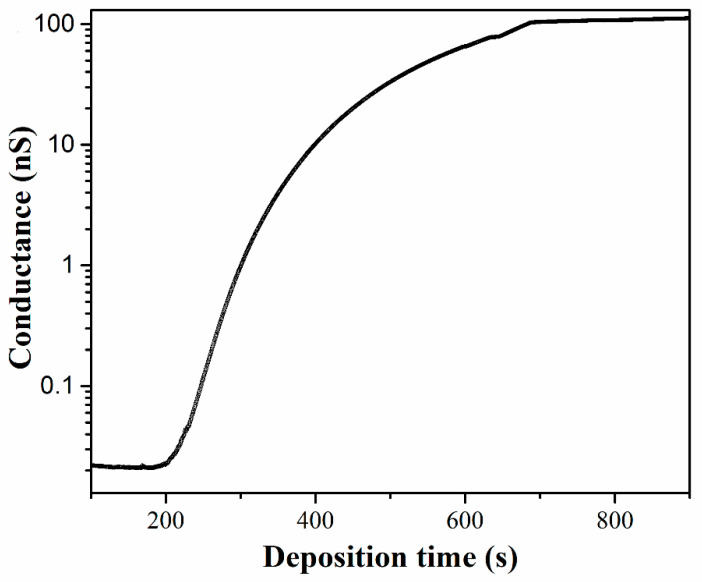
Electron conductance of the Pd NP film monitored during the NP deposition process.

The evolution of the morphology and electron conductance of the NP films during NP deposition is examined. Figure 3 shows the conductance evolution of the Pd NP film measured simultaneously with the deposition process. At the earlier stage of the deposition, the coverage of the NPs on the surface is too low to generate any electron conduction across the electrodes, therefore, no conductance can be measured. After 185 s of deposition, with an equivalent 7.4 nm Pd being deposited, a conductance which is distinguishable with the leakage conductance of the substrate starts is observed. This means the NP coverage is sufficiently high to generate electron conduction paths in the NP film distributed across the electrodes. Between 200 and 350 s, the conductance displays a dramatic increase by two orders of magnitude, growing approximately exponentially with the deposition time. After that, the rising of the conductance becomes slow and finally tends to plateaus, generating another two orders of magnitude increase within the next 360 s deposition. When a total deposition time of 690 s is reached, the deposition is stopped by operating a shutter. The conductance then remains stable at about 100 nS, which is four orders of magnitude larger than the leakage conductance of the substrate.

**Figure 4:**
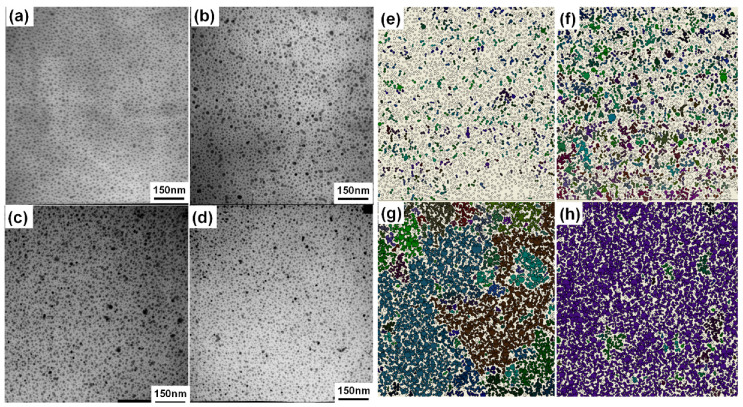
TEM images of the Pd NP films with deposition time of (**a**) 100 s, (**b**)175 s, (**c**) 350 s and (**d**) 650 s and the corresponding digitalized NP location mapping (**e**–**h**), with each percolation cluster being marked with different colors.

Four specimens for TEM characterization are collected during the NP deposition stage either before or after a measurable electron conductance can be observed. The deposition time of these specimens is 100 s, 175 s, 350 s and 650 s, respectively. TEM microimages of these specimens are shown in Figure 4. The surface coverages of the Pd NPs measured from Figure 4a–d are 28%, 40%, 62% and 79%, respectively.

Considering there is a direct correspondence between the NP coverage and the deposition time, the time-dependent conductivity curve shown in Figure 3 is indicative of the percolation character in NP films [26,27]. The NP deposited film can be simply described with a two-dimensional network, where a random fraction of the interconnections is occupied. The number, size and shape of the connected structures can be explained using percolation theory [28]. At a defined coverage the percolation threshold is approached and the NP network becomes conducting over the length scale defined by the separation of the electrodes. On the other hand, it has been shown in previous studies that the electron conduction in metal NP arrays is proceeded by tunneling or hopping between neighboring NPs [29,30]. The tunneling conductance between adjacent nanoparticles depends exponentially on the separation gap between them. We thus set a critical separation gap permitting electron conduction and used the percolation approach to analyze the development of the electron conductance of the NP films during the deposition process. Assuming a critical separation gap of 1.2 nm, we searched the percolation clusters from the digitalized TEM images by mapping the NPs onto two-dimensional networks composed of electrically interconnected NPs. The arrangements of the percolation clusters in the NP films are shown in Figure 4e–h. In the figure, each percolation cluster is indicated with different colors.

From Figure 4, we can find that when the NP coverage is low, such as that in Figure 4a with a coverage of 28%, the NP arrays are mainly composed of electrically separated particles. Only a few percolation clusters of very small size, containing less than 10 NPs, can be identified, as shown in Figure 4e. Obviously, there is no percolation pathway for electron conduction across the upper edge and the bottom edge of the image. With the increase of the NP coverage, the size and the density of the percolation clusters increases significantly, as shown in Figure 4f. In Figure 4c, the NP coverage becomes sufficiently larger (62%), a few large percolation clusters connecting the upper edge and the bottom edge of the image appear, as shown in Figure 4g. An NP film with sufficient electron conductivity is thus formed. However, there still remains a number of relatively smaller percolation clusters, which can potentially grow into percolation pathways for electron conduction. Therefore, the conductance displays a dramatic increase with further NP deposition. In Figure 4d, the NP coverage becomes so high (79%) that most of the NPs are composed in a huge percolation cluster expanding over the whole image (Figure 4h). Although a few small percolation clusters embedded in the huge percolation cluster can still be identified, they will merge into the existing huge one rather than grow into a separate percolation pathway under further NP deposition and therefore contribute little to the increase of the electron conductance. As a result, the conductance increases slowly and approaches plateaus under further NP deposition.

**Figure 5:**
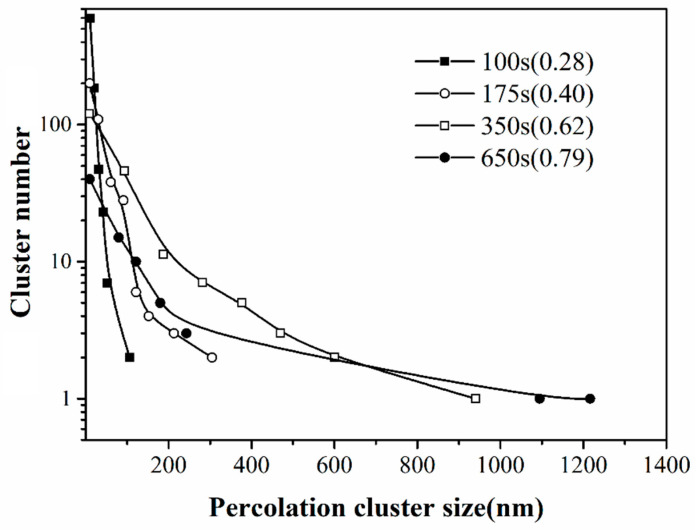
Size distributions of the percolation clusters analyzed from the TEM images of the Pd NP films with deposition time of 100 s, 175 s, 350 s and 650 s. The coverage of the NP films is shown in the corresponding legend.

In Figure 5, the size distributions of the percolation clusters are compared for the NP films with different deposition times. In every coverage, the number of the percolation clusters decreases rapidly with the cluster size. With the increase of the coverage, both the total number of percolation clusters and the number of smaller clusters sufficiently decrease, accompanied with the formation of a small number of larger clusters. This indicates that more and more smaller percolation clusters are merged into larger ones with the ongoing cluster deposition. The largest size of the percolation clusters increases significantly with the NP coverage, however, their number always remains low. Therefore, in a network system formed by random deposition of particles, there is no way to generate large size percolation clusters in high density. This means the electron conductance of the NP arrays is contributed to by a limited number of percolation pathways for electron conduction.

**Figure 6:**
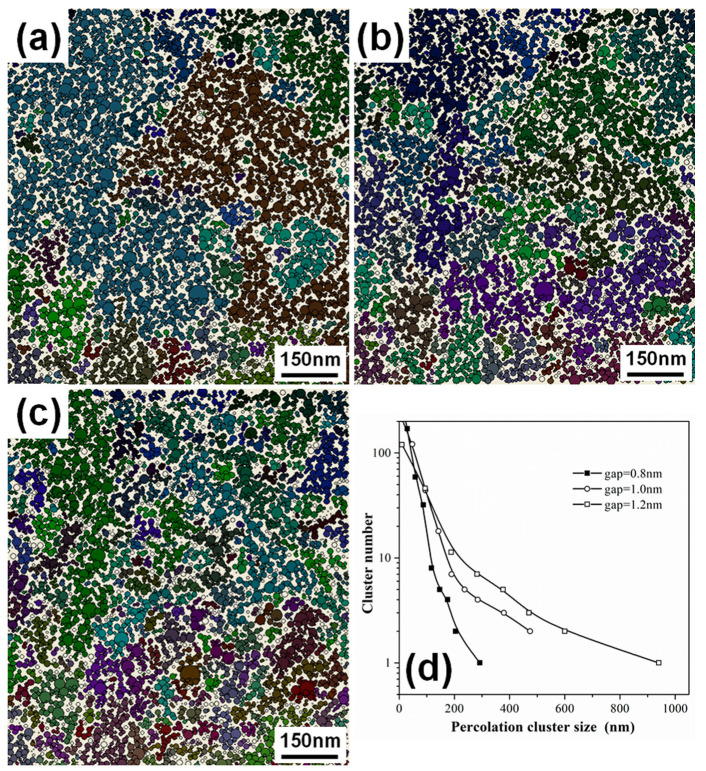
Digitalized NP location mappings generated from the TEM image of the Pd NP film with deposition time of 350 s. The percolation clusters are searched by assuming a critical separation gap for electron conduction of (**a**) 0.8 nm, (**b**) 1.0 nm and (**c**) 1.2 nm, respectively. Each percolation cluster is marked with different colors. (**d**) Size distributions of the percolation clusters in (**a**–**c**).

In Figure 6, the arrangements of the percolation clusters in the NP films with 62% coverage, mapped by assigning a critical separation gap for electron conduction of 0.8 nm, 1.0 nm and 1.2 nm, are shown, together with the size distributions of the percolation clusters. By using a smaller critical separation gap, the larger percolation clusters split into a number of smaller ones, and the size distribution curve shifts to a smaller size region. Meanwhile, the size of the largest percolation cluster decreases significantly. This means the number of percolation pathways for electron conduction reduces if the critical separation gap for electron conduction becomes smaller. Reducing the critical separation gap in the percolation analysis is equivalent to the effect of expanding the NP arrays, therefore, the number of percolation pathways for electron conduction can be sufficiently changed via a strain applied on the NP arrays.

In the above image analyses, the length scale of the percolation system is about 100, as normalized to the NP size. Although this length is smaller than the separation between the electrodes used for conductance measurement (with a normalized length of 1500), it is sufficiently large enough to ensure that there is not significant difference on the percolation threshold between the two systems, since according to the percolation theory of finite system [31], the percolation threshold changes little with the system size when the normalized system size is larger than 100. Therefore, it is reasonable to assume that the above percolation nature analyzed from the TEM images is applicable to the real system used in the electron conduction measurements.

**Figure 7:**
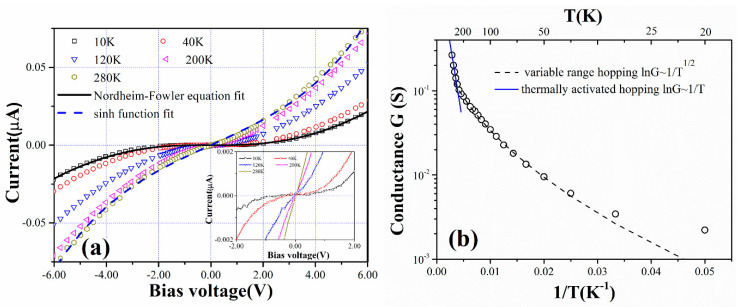
(**a**) The I-V curves of the 62% coverage Pd NP film measured at different temperatures. The solid curve and dashed curve fit the experimental data with a Nordheim-Fowler equation and sinh function, respectively. The inset shows a magnified view to illustrate the blockade characteristics at cryogenic temperatures. (**b**) Temperature dependences of the zero bias conductance. The solid curve and dashed curve fit the experimental data with lnG ~ 1/T scaling and lnG ~ 1/T^1/2^ scaling, respectively.

In order to understand the electron transport characteristics in the Pd NP films, the current–voltage (I–V) curves are measured from 20 K to room temperature. The measurement is carried out on a pair of IDEs deposited with Pd NPs with a coverage of 62%. As shown in Figure 7a, at cryogenic temperatures the I–V curves display a current plateau with low applied bias voltages, demonstrating Coulomb blockade behavior. The I–V curve at 20 K can be well fitted by the Nordheim-Fowler equation [32], indicating tunnel emission is the dominate transport mechanism at this temperature. With the increase of the temperature, the I–V characteristic becomes more and more linear, displaying a smooth transition from the blocked regime to the unblocked regime. At room temperature, no current plateau is observable, but the I–V curve is still distinctly nonlinear. The I–V curves could be well fitted by a sinh function, suggesting electron hopping becomes the dominate transport mechanism [33]. To get a deeper insight into the charge transport mechanism, we deduced zero bias conductance (G) from the I-V curves measured at different temperatures. Figure 7b shows the temperature dependence of the conductance. A significant conductance increase is observed with the increase of temperature, indicating that the electron transport in the NP film is strongly different from the normal metallic films. In fact, the positive temperature coefficient of conductance is indicative of thermally activated transport. In Figure 7b, from room temperature to ∼200 K, ln(G) scales as 1/T, indicating that thermally activated hopping dominates the transport in this temperature regime [22]; electron transfer occurs between spatially adjacent NPs. At lower temperature, deviation from the 1/T scaling occurs, ln(G) scales as T^1/2^, indicating that variable range hopping becomes the dominant electron transport mechanism [21]; electron transfer occurs between the energetically closest NP states. For temperatures lower than 50 K, the conductance can be fitted neither with 1/T scaling nor with T^1/2^ scaling, indicating that thermally activated hopping no longer dominates the electron transport. In this regime, electrons are tunneling when the applied bias voltage is sufficient to overcome the charging energies of the NPs. The electron transport behavior observed above is analogous to previous studies on two-dimensional NP arrays with well-defined inter-particle separation gaps [20,22,34], suggesting that the electron conductance in the fabricated NP films is proceeded by quantum transport dominated by tunneling and/or hopping and is subject to a Coulomb blockade at moderate voltages.

**Figure 8:**
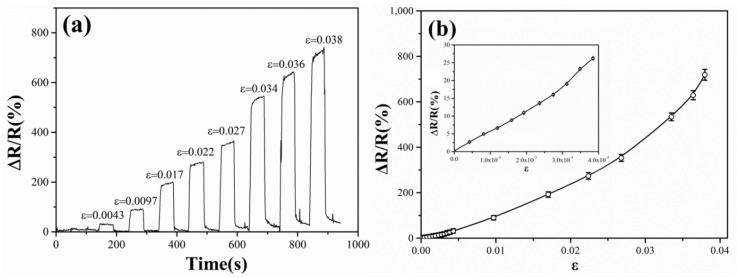
(**a**) Electrical resistance response curve of the strain sensor under pull-and-release cycles. (**b**) Relative resistance changes as a function of the applied strains. The insert illustrates the response behavior to very small strains generated by subjecting the PET membrane to bending cycles.

The Pd NP-coated PET membranes are used as strain sensors and their strain sensing responses are studied at room temperature. In Figure 8a, the measured conductance response in the pull-and-release cycles of the PET membrane is given as the relative resistance variation ΔR/R (where ΔR is the resistance change induced by deformation and R is the resistance without strain). The specimen under testing is a 62% coverage Pd NP film deposited on an IDE patterned PET membrane with 0.1 mm thickness. The curve shows that for each pull-and-release cycle the resistance increases quickly, which corresponds to the decrease of the conductance of the NP film, once the device is stretched, and returns to the original value instantly once the applied stress is released. The strain sensing response is rapid, reversible and reproducible. The stretching of the PET membrane induces a strain on the surface where the NPs deposit. The inter-particle separation gaps in the NP film thus increase, which results in an increase on the resistance of the NP film.

Figure 8b shows the sensor response plotted as a function of the applied strains. The response is taken as the peak resistance variation ΔR/R, after the PET membrane is pulled to a stable state. In the inset of Figure 8b, the sensor response curve is magnified to illustrate the response behavior to very small applied strains. Such small strains are generated by subjecting the PET membrane to bending cycles, which induce stretching strains on the surface where the NPs deposit. In either case, the resistance increases with the applied strain sensitively. As can be seen from the figure, the sensor can distinguish tiny strain changes down to 5 × 10^−4^. In the small applied strain region (<0.5%), the strain sensor displays a constant sensitivity, which is characterized with the gauge factor g = (ΔR/R)/ε. The gauge factor is calculated to be ~55 in this strain region. The constant gauge factor enables a linear response of the relative resistance changes to the applied strains. In the moderate strain region, from 1.0% to 4.0%, a high gauge factor is maintained, between 100 to 200, and increases gently with the applied strains. The sensor can reversibly respond to applied strains up to 4.0%. However, this upper limitation comes from the elastic limit of the PET membrane. It is possibly extended significantly if other more elastic materials, such as PDMS (polydimethylsiloxane), are used.

The behavior of the NP film-based strain sensor can be understood by considering the percolative and quantum tunneling nature of the closely-spaced NP arrays. The IDEs we used have a huge aspect ratio of total electrode length versus electrode separation. With a moderate NP coverage above the percolation threshold corresponding to the finite length of the electrode separation [31], a large number of percolative paths are contained in the NP arrays. Since the tunneling conductance between adjacent NPs depends exponentially on the inter-particle separation gap, the number of percolative paths for electron conduction could be changed by a tiny deformation in the geometries of the NP arrays, which induces breaking or regenerating of the percolation clusters. Furthermore, the deformation also induces a change on the distribution of inter-particle gaps along the conductive percolation pathways, which also influence the tunneling conductance sensitively. As a result, the conductance of percolative NP arrays is sensitively related to the deformation of the substrate on which the NPs deposit.

## 4. Conclusions

Gas phase NP beam deposition, as a low-cost and semiconductor device-compatible process, is used to fabricate closely spaced NP arrays on flexible membranes patterned with IDEs. The evolution of the morphology and electron conductance of the NP films during deposition is examined by performing real-time conductance measurement, TEM characterization and image analysis. It is shown that the NP film is composed of two-dimensional percolation clusters of interconnected NPs. The conductance of the NP film is contributed to by a limited number of percolation pathways for electron conduction. Both the percolation geometries and conductance of the NP film are determined by the NP coverage, which is finely controlled by the deposition time under a constant deposition rate. This permits us to finely control the NP deposition process to realize a precise and reproducible fabrication of sensing materials for strain and deformation gauging. Electron transport measurement reveals that the electrical conductance of the NP films is dominated by electron tunneling and hopping across the NP percolating networks. Since the tunneling conductance between adjacent NPs depends exponentially on the inter-particle separation gap, the number of percolative paths for electron conduction and the distribution of inter-particle gaps along the conductive percolation pathway could be changed by a tiny deformation in the geometries of the NP arrays, resulting in a sensitive influence on the tunneling conductance. Based on the percolative and quantum tunneling nature, the closely-spaced Pd NP films on PET membranes are used as sensing materials for flexible strain sensors, which demonstrate an excellent ability to distinguish tiny strain changes down to 5 × 10^−4^ and a high sensitivity with a large gauge factor of 200 up to 4% applied strain.
